# Retraction Note: Osteolytic bone metastasis is hampered by impinging on the interplay among autophagy, anoikis and ossification

**DOI:** 10.1038/s41419-022-04991-7

**Published:** 2022-06-09

**Authors:** P. Maroni, P. Bendinelli, E. Matteucci, A. Locatelli, T. Nakamura, G. Scita, M. A. Desiderio

**Affiliations:** 1 Istituto Ortopedico Galeazzi, IRCCS, Milan, Italy; 2 Dipartimento di Scienze Biomediche per la Salute, Molecular Pathology Laboratory, Università degli Studi di Milano, Milan, Italy; 3 Istituto FIRC di Oncologia Molecolare, Milan, Italy; 4 Center for Advanced Science and Innovation, Osaka University, Yamadaoka 2-1, Osaka, 565-0871 Japan; 5 Dipartimento di Scienze della Salute, Università degli Studi di Milano, Milan, Italy

Retraction to: *Cell Death and Disease* 10.1038/cddis.2013.465, published online 16 January 2014

The Editors are retracting this article because of concerns with a number of figures. An investigation by the University of Milan confirmed that:

- The vinculin bands in lanes 1–2 in Fig. 8b are used to represent vinculin in a different experiment published as Fig. 4A lanes 1–2 in [1].
- In Fig. 8d the Bim EL, BimL and BimS bands have been duplicated in lanes 4 & 5.
- In Fig. 8d the p-Akt bands in lanes 4 & 5 have been duplicated.

The Editors therefore no longer have confidence in the data.

Maria Alfonsina Desiderio does not agree to this retraction. Paola Maroni, Paola Bendinelli, Emanuela Matteucci, Alessia Locatelli and Giorgio Scita have not responded to any correspondence from the editor/publisher about this retraction. The publisher was unable to find up-to-date contact details for Toshikazu Nakamura.
